# Acceptance sampling plan based on difference in difference estimator with application

**DOI:** 10.1038/s41598-023-49786-8

**Published:** 2023-12-15

**Authors:** Muhammad Azam, Maira Ahsan Khan, Asma Arshad, Muhammad Saleem, Muhammad Aslam

**Affiliations:** 1 Department of Statistics and Computer Science, University of Veterinary and Animal Sciences, Lahore, 54000 Pakistan; 2 Department of Statistics, Kinnaird College for Women, Lahore, Pakistan; 3 National College of Business Administration and Economics, Lahore, 54000 Pakistan; 4 Department of Industrial Engineering, Faculty of Engineering-Rabigh, King Abdulaziz University, 21589 Jeddah, Saudi Arabia; 5 Department of Statistics, Faculty of Science, King Abdulaziz University, 21551 Jeddah, Saudi Arabia

**Keywords:** Materials science, Nanoscience and technology

## Abstract

An acceptance sampling plan has been designed in this study based on the Difference-in-Difference estimator. This plan is designed for the inspection of those product units whose life follows the normal distribution. The operating characteristic function is discussed for the two respective cases of the standard deviation known and unknown. The parameters of the proposed plan are determined by minimizing the sample size and followed by the satisfying optimization rule. The results are computed and tabulated for various parametric combinations of acceptable quality levels and limiting quality levels. The computations are performed by using R statistical programming software for all respective cases. The real-life application of the proposed sampling plan has been discussed and elaborated in detail.

## Introduction

An essential component of industrial production is the process of inspecting the finished goods through acceptance sampling plans after they are manufactured, as these plans are characterized by lot sentencing procedures. Dealing with these sampling plans is crucial since they will offer a better method for determining the minimum sample size in each scenario for lot sentencing and whether it is worthwhile to be accepted or rejected. To preserve or assert a certain level of reputation regarding the manufacturing lot, the industries are susceptible to passing through their ready or final production lots through suitable acceptance sampling plans^1–4^. The field of statistical process control (SPC) offers tools such as acceptance sampling plans that are better suited for a range of industrial settings^5^. In this situation, producers want to favor the minimum sample size acceptance sampling plans since they are eager to prevent needless inspection expenses and effort^6^. As a result, plans that align with production conditions are implemented. Producers are constantly interested in using effective, remarkable sample plans that need less inspection time and money to decrease losses and maintain high standards of quality^7^.

Similar to how effective sampling plans in production lines are crucial for quick lot sentencing, acceptance-sampling plans are among the most critical instruments for this purpose^6^. Plans come in two varieties: variable acceptance sampling plans and attribute plans. The literature on SPC is highly comprehensive and offers suitable acceptance sampling plans tailored to the requirements of the production settings. Earlier ones are thought to have easier industrial implementations among them. However, today's producers are more picky, using more data and being more sensitive to achieve the desired outcomes; for this reason, variable sampling plans are preferred over attribute sampling plans^8^. Since extraneous factors naturally disrupt manufacturing, there are two risks associated with lot sentencing that cannot be avoided: accepting the good lot and rejecting the bad one^7^. There are numerous real-world situations when having some extra, antecedent, or auxiliary knowledge can significantly enhance the decision-making process regarding lot sentencing methodology. Quality control literature favors the utilization of additional information in support of the study variable $y$.

As^8^ utilized the single auxiliary variable in designing of acceptance sampling plan and successfully achieved improved results. The literature on statistical process monitoring control charts by using a single auxiliary variable is also quite enriched. A detailed discussion of this concept can be seen in^9–14^ and^15^. As^16^ presented auxiliary information-based arithmetic mean control chart^17^. Proposed auxiliary information EWMA control chart^18^. Developed the use of multivariate regression-based mean and variance control charts^19^. Constructed an individual monitoring control chart that is based on cluster-regression methods. Recently^20^, introduced the concept of auxiliary information to enhance the more proficient way of an acceptance sampling plan to accept the lot^21^, designed the economical way of an acceptance sampling plan despite having inspection errors^22^, utilized the regression estimator concept in developing the EWMA statistic based acceptance sampling plan^23^, employed the regression estimator in the presence of uncertainty to create a successful plan^24^, suggested repeated sample acceptance sampling strategy based on multiple dependent state sampling, both with and without the use of an auxiliary dataset to demonstrate the effectiveness of the suggested plan^25^, recommended product acceptance by taking into account the liner profiles of two suppliers using Wang's test statistic to determine the required minimum sample size to obtain an effective plan. Moreover^26–34^, and^35^ introduced more of this kind of concept procedure, which works well under certain production environments.

The new contribution of the presented research is the development of a new acceptance sampling plan when more than one auxiliary variable is demanded by both the producer and the consumer to estimate the study variable or the variable of interest in a much more precise manner to satisfy the risks respectively. The new index with three auxiliary variables used in this paper methodology is novel, and the use of the difference-in-difference estimator approach produces better results in terms of the smaller sample size needed to make a better decision of lot sentencing than the other existing acceptance sampling plans now in literature. To the best of the authors' knowledge, no attempt has been made to create an acceptance sampling plan in the literature. This leads to the creation of the suggested study. The difference estimator plays a significant role in the statistical literature, particularly in situations where the goal is to estimate the difference between two population parameters. Here are some contributions of the concept in statistical process control as acceptance sampling plans and examples as follows: Treatment Effects in Experimental Studies: Difference estimators are commonly used to assess treatment effects in experimental studies. They help quantify the impact of a treatment or intervention by comparing the outcomes. Example: In a clinical trial, a new drug's effectiveness is evaluated by measuring the difference in health outcomes between patients who received the drug and those who received a placebo to make a decision about the effectiveness of treatment. Before-and-After Studies: Difference estimators are valuable in assessing changes over time, as they quantify the difference in outcomes before and after an intervention or policy change. Example: A city implements a traffic management system, and the difference in traffic congestion levels before and after the system's implementation is used to estimate the system's impact. Paired Observations and Matched Samples: Difference estimators are commonly employed when dealing with paired observations or matched samples. They help account for individual variability by focusing on the differences within pairs or matched groups. Example: In a study comparing the effectiveness of two teaching methods, the difference in test scores for each student is calculated, and the average difference is used as the estimator. Economic Studies—Control Groups: Difference estimators are essential in economic studies, especially when dealing with observational data. They help control for unobserved factors by comparing differences in outcomes over time or between groups. Example: Evaluating the impact of a policy change on employment rates by comparing the differences in employment levels before and after the policy change. Quasi-Experimental Designs: In situations where true randomization is challenging, difference estimators are valuable in quasi-experimental designs, helping to control for confounding variables. Example: Assessing the impact of a new teaching method in a school where random assignment is not feasible, by comparing the difference in performance between classes that adopted the new method and those that did not. In summary, the difference estimator contributes to estimating and interpreting the impact of interventions, policy changes, or experimental conditions by quantifying the differences in outcomes, making it a versatile tool in various fields of study.

**Figure 1:**
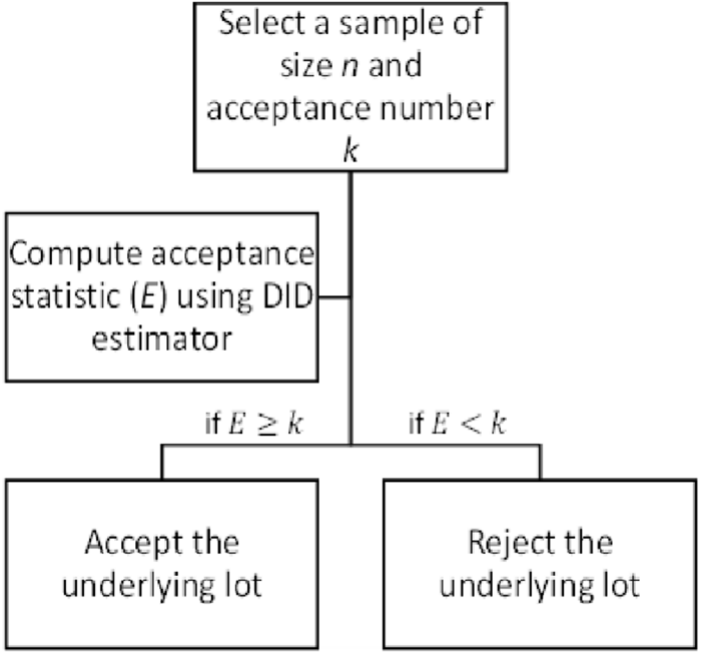
Flow Chart of the Proposed DID Sampling Plan Methodology.

To develop an acceptance sample plan, we have incorporated in this study the use of two auxiliary variables in addition to the variable of interest. No acceptance sample strategy is created with two auxiliary variables, according to the authors' information. Thus, the suggested methodology, which was first put forth by^36^, uses the auxiliary data in the form of a Difference-in-difference (DID) estimator to examine the variable of interest. The minimum necessary lot inspection sample size is used to assess the proposed concept's competency. The remaining portions of the paper are divided as follows: The conceptual foundation of the DID estimator and the suggested plan's approach are covered in Sections "Methods" and "Results discussion". The real-world example and the determined findings from the simulation runs are described in Section "Results findings", and the concluding observations are contained in Section "Conclusions". Figure 1 displays the methodology's flowchart, while Table 1 lists the key symbols and notations before the Section "Introduction" introduction.

**Table 1:** Symbols and Notations.

EWMA	Exponentially Weighted Moving Average
OC	Operating Characteristic
${\overline{Y} }_{DID}$	Population Mean through DID Estimator
$\overline{y }$	Sample Mean of Variable $y$
${\mu }_{y}$	Population Mean of Variable $y$
${\mu }_{x}$	Population Mean of Variable $x$
$\overline{x }$	Sample Mean of Variable $x$
${\mu }_{z}$	Population Mean of Variable $z$
$\overline{z }$	Sample Mean of Variable $z$
${\beta }_{yx}$	Regression Coefficient between $y$ and $x$
${\beta }_{xz}$	Regression Coefficient between $x$ and $z$
${\beta }_{yz}$	Regression Coefficient between $y$ and $z$
${\sigma }_{x}^{2}$	Population Variance of variable $x$
${\sigma }_{y}^{2}$	Population Variance of variable $y$
${\sigma }_{z}^{2}$	Population Variance of variable $z$
${\rho }_{yx}$	Population Correlation between Variable $y$ and $x$
${\rho }_{yz}$	Population Correlation between Variable $y$ and $z$
${\rho }_{xz}$	Population Correlation between Variable $x$ and $z$
$E$	Acceptable Approved Statistic
$k$	Acceptance Number
$USL$	Upper Specification Limits
${z}_{p}$	${p}^{th}$ Percentile of the Norma Distribution
$\Phi (.)$	Cumulative Density Function of Standard Normal Distribution
AQL	Acceptable Quality Level
LQL	Limiting Quality Level
$\alpha$	Producer’s Risk
$\beta$	Consumer’s Risk
$S$	Sample Standard deviation of $n$ values of variable $y$
${y}_{i}$	Ith Observations of Sample $y$ values
${c}_{4}$	A Constant

## Methods

In this section, the procedures opted to be discussed for the designs of the proposed sampling plan are mentioned here. The underlying problem statement and strength that need to be addressed are as follows:

At times, the producers seem it complicated to change the sampling schemes with a complex one than simply the simple random sampling scheme, and also the producers believe that various auxiliary information is readily available as part of the production system to use them in improving the precision of the study variable estimate than the usage of complex sampling schemes. This philosophy provides a better understanding of an acceptance sampling scheme as far as the utilization of various auxiliary variables in the estimation does not hamper the sampling cost and gives much-improved results to define defectives as defective items as well.

The proposed acceptance sampling plan is based on the DID estimator introduced by^36^, as follows:

1 $${\overline{Y} }_{DID}= \overline{y }+\frac{1}{1-{\rho }_{xz}^{2}} {\beta }_{yx}\left[{\mu }_{x}- \left\{\overline{x }+ {\beta }_{xz}\left({\mu }_{z}- \overline{z }\right)\right\}\right]+\frac{1}{1-{\rho }_{xz}^{2}} {\beta }_{yz}\left[{\mu }_{z}- \left\{\overline{z }+ {\beta }_{zx}\left({\mu }_{x}- \overline{x }\right)\right\}\right]$$

The variable $y$ is the study variable while $x$ and $z$ are the auxiliary variables. There are $l$ random samples drawn for each variable $\left\{\left({y}_{ij,}{x}_{ij,}{z}_{ij}\right): i=\mathrm{1,2},3,\dots ,l; j=\mathrm{1,2},3,\dots ,n\right\}$ with a sample of size $n$ and taken as the tri-variate normal random variable which follows the normal distribution having mean $\underset{\_}{\mu }$ as a vector of $l$ values given as under:

2 $$\underset{\_}{\mu }=\left[\begin{array}{c}{\mu }_{y}\\ {\mu }_{x}\\ {\mu }_{z}\end{array}\right]$$

and the variance–covariance matrix $\sum_{\_}$ concerning the corresponding $y,x,$ and $z$ variables are as follows:

3 $$\sum_{\_}=\left[\begin{array}{ccc}{\sigma }_{y}^{2}& {\rho }_{yx}{\sigma }_{y}{\sigma }_{x}& {\rho }_{yz}{\sigma }_{y}{\sigma }_{z}\\ {\rho }_{yx}{\sigma }_{y}{\sigma }_{x}& {\sigma }_{x}^{2}& {\rho }_{xz}{\sigma }_{x}{\sigma }_{z}\\ {\rho }_{yz}{\sigma }_{y}{\sigma }_{z}& {\rho }_{xz}{\sigma }_{x}{\sigma }_{z}& {\sigma }_{z}^{2}\end{array}\right]$$

Here, ${\mu }_{y},{\mu }_{x}$ and ${\mu }_{z}$ are the population means of $y, x,$ and $z$ for the $l$ terms and ${\sigma }_{y}^{2},{\sigma }_{x}^{2}$ and ${\sigma }_{z}^{2}$ are the population variances, while ${\rho }_{yx}$, ${\rho }_{yz}$ and ${\rho }_{xz}$ are the possible correlation coefficients between the respective three variables. The mean and variance of the $DID$ estimator are given as:

4 $$E\left({\overline{Y} }_{DID}\right)={\mu }_{y}$$

and

5 $$Var\left({\overline{Y} }_{DID}\right)=\frac{1}{n\left(1-{\rho }_{xz}^{2}\right)} {\sigma }_{y}^{2}\left[1-{\rho }_{yx}^{2}-{\rho }_{yz}^{2}-{\rho }_{xz}^{2}+{2\rho }_{yx}{\rho }_{yz}{\rho }_{xz}\right]$$

The acceptance sampling plan that has been suggested is for the two cases of σ known and unknown, which are examined independently as follows:

**Case 1** In σ known circumstances.

In terms of the single sampling-based acceptance sampling plan, the suggested sampling plan design implements the^36^ in statistical process control. It is suggested to use the following operating characteristic (OC) function with a smaller sample size (*n*) than the typical sampling schemes currently in use:

6 $$L\left(p\right)=P\left(E\ge k\right)$$

Here, $E$ is the value of the acceptable, approved statistic provided as:

7 $$E=\frac{USL-{\overline{Y} }_{DID}}{{\sigma }_{y}}$$

Here, $k$ is the acceptance number that will be ascertained via simulation runs, and $USL$ is the upper specification limit set by the manufacturer.

## Design

The following are the steps involved in implementing the suggested sample design:

**Step 1** Ascertain the critical risk values for producers and consumers, denoted as $\alpha$ and $\beta$, in addition to $USL$. Compute the acceptance statistic $E$ using a tri-variate simple random sample of size $n$ from the lot.

**Step 2** Take the following stance on the lot's disposition::

1. If $E\ge k$, accept the inspected lot.
2. If $E<k$, reject the inspected lot.

The OC function becomes

8 $$L\left(p\right)=P\left(\frac{USL-{\overline{Y} }_{DID}}{{\sigma }_{y}}\ge k\right)$$

As per^37^, it is assumed that if the population under study follows the normal distribution then the distribution of the ${\overline{Y} }_{DID}$ will also follow the normal distribution.

Hence, it can be shown as

9 $${\overline{Y} }_{DID}+k{\sigma }_{y}\sim N\left({\mu }_{y}+k{\sigma }_{y},\frac{1}{n\left(1-{\rho }_{xz}^{2}\right)} {\sigma }_{y}^{2}\left[1-{\rho }_{yx}^{2}-{\rho }_{yz}^{2}-{\rho }_{xz}^{2}+{2\rho }_{yx}{\rho }_{yz}{\rho }_{xz}\right]\right)$$

The probability of acceptance is:

10 $$L\left(p\right)=\Phi \left(\frac{{z}_{p}-k}{\sqrt{\frac{1}{n\left(1-{\rho }_{xz}^{2}\right)} {\sigma }_{y}^{2}\left[1-{\rho }_{yx}^{2}-{\rho }_{yz}^{2}-{\rho }_{xz}^{2}+{2\rho }_{yx}{\rho }_{yz}{\rho }_{xz}\right]}}\right)$$

The ${z}_{p}$ and $\Phi (.)$ represents the ${p}^{th}$ percentile and the cumulative density function (CDF) following the standard normal distribution.

Rejecting a good lot and accepting a bad lot are the two risks associated with the lot sentencing procedure that are present in the acceptance sampling strategy. Let's designate $\alpha$ as the producer's risk and $\beta$ as the consumer's risk. We should also designate ${p}_{1}$ as the acceptable quality level (AQL) and ${p}_{2}$ as the limiting quality level (LQL). The suggested plan is based on certain plan parameters that were found in the simulation process' output so that the two connected points ($\alpha ,{p}_{1}$) and ($\beta ,{p}_{2}$) pass through the center of the curve end to end and satisfy the subsequent two-fold optimization procedures with the smallest possible sample size $(n)$.

As long as the following criteria are met:

11 $$L\left({p}_{1}\right)=\Phi \left(\frac{{z}_{{p}_{1}}-k}{\sqrt{\frac{1}{n\left(1-{\rho }_{xz}^{2}\right)} {\sigma }_{y}^{2}\left[1-{\rho }_{yx}^{2}-{\rho }_{yz}^{2}-{\rho }_{xz}^{2}+{2\rho }_{yx}{\rho }_{yz}{\rho }_{xz} \right]}}\right)\ge 1-\alpha $$

and

12 $$L\left({p}_{2}\right)=\Phi \left(\frac{{z}_{{p}_{2}}-k}{\sqrt{\frac{1}{n\left(1-{\rho }_{xz}^{2}\right)} {\sigma }_{y}^{2}\left[1-{\rho }_{yx}^{2}-{\rho }_{yz}^{2}-{\rho }_{xz}^{2}+{2\rho }_{yx}{\rho }_{yz}{\rho }_{xz} \right]}}\right)\le \beta $$

Additionally, we set the correlation coefficient values to ${{{\rho }_{xz}=\rho }_{xy}=\rho }_{yz}=\rho$. The observed trend between the computed values is examined in the following manner based on Table 2:

1. For the fixed values of ${p}_{1}$, sample size $n$ decreases as ${p}_{2}$ increases. For instance, ${p}_{1}= 0.001$ at $\rho =0.7$ for ${p}_{2} = 0.004,$ $n=20; \mathrm{for }{p}_{2} = 0.006, n=11$*;* for ${p}_{2} = 0.008,$ $n=8$. But $n = 4$ for ${p}_{2}= 0.020$.
2. For the fixed values of ${p}_{2}$, sample size $n$ increases as ${p}_{1}$ decreases. For instance, ${p}_{2}= 0.020$ at $\rho =0.7$ for ${p}_{1} = 0.001,$ $n=4; \mathrm{for }{p}_{1} = 0.0025, n=7$*;* for ${p}_{1} = 0.005,$ $n=14$. But $n = 49$ for ${p}_{1}= 0.010$.
3. As the value of the $\rho$ decreases the value of sample size increases. For instance, at ${p}_{1}= 0.001$ and ${p}_{2} = 0.006$ at $\rho =0.9$ the $n=2; \mathrm{at }\rho =0.7\mathrm{ the } n=11$ and $\rho =0.5$ the $n=18.$

**Table 2:** Plan parameters of single sampling plan using DID estimator when $\sigma$ is known at different values of ${{{\rho }_{xz}=\rho }_{xy}=\rho }_{yz}=\rho$.

${p}_{1}$	${p}_{2}$	$\rho =0.9$		$\rho =0.8$		$\rho =0.7$		$\rho =0.6$		$\rho =0.5$		$\rho =0.4$		$\rho =0$	
		*n*	*k*	*n*	*k*	*n*	*k*	*n*	*K*	*n*	*k*	*n*	*k*	*n*	*k*
0.001	0.002	15	2.97	57	2.97	83	2.97	106	2.97	128	2.97	149	2.97	197	2.97
	0.003	6	2.89	22	2.9	32	2.9	42	2.89	49	2.9	57	2.9	74	2.9
	0.004	4	2.83	13	2.84	20	2.85	25	2.84	31	2.85	35	2.84	45	2.84
	0.006	2	2.76	8	2.77	11	2.76	15	2.77	18	2.77	20	2.77	26	2.76
	0.008	2	2.75	6	2.71	8	2.7	11	2.71	13	2.7	15	2.7	19	2.71
	0.01	2	2.73	5	2.67	7	2.66	9	2.66	10	2.66	12	2.65	15	2.66
	0.015	2	2.54	3	2.58	5	2.58	6	2.57	7	2.57	8	2.57	11	2.57
	0.02	2	2.76	3	2.56	4	2.54	5	2.54	6	2.51	7	2.54	8	2.51
0.0025	0.005	13	2.68	48	2.68	69	2.68	89	2.68	108	2.68	125	2.68	163	2.68
	0.01	3	2.54	11	2.54	16	2.54	21	2.53	25	2.54	29	2.54	39	2.53
	0.015	1	2.48	7	2.46	9	2.45	12	2.45	15	2.44	17	2.44	22	2.44
	0.02	2	2.42	5	2.38	7	2.38	9	2.39	11	2.37	12	2.39	16	2.39
	0.025	2	2.27	4	2.36	6	2.36	7	2.33	9	2.32	10	2.35	12	2.33
	0.03	2	2.45	3	2.29	5	2.29	6	2.3	7	2.29	8	2.3	11	2.29
	0.05	2	2.4	2	2.15	3	2.14	4	2.16	5	2.18	5	2.16	7	2.17
0.005	0.01	11	2.43	43	2.43	59	2.44	77	2.44	93	2.44	109	2.43	139	2.44
	0.015	4	2.35	16	2.35	23	2.35	30	2.35	35	2.35	41	2.35	53	2.35
	0.02	3	2.27	10	2.29	14	2.29	18	2.28	22	2.28	25	2.28	32	2.28
	0.03	2	2.22	6	2.19	8	2.18	10	2.19	12	2.18	14	2.18	18	2.18
	0.04	2	2.01	4	2.13	6	2.1	7	2.11	9	2.12	10	2.11	13	2.11
	0.05	2	2.04	3	2.06	5	2.04	6	2.03	7	2.05	8	2.06	10	2.06
	0.1	2	1.91	2	1.92	3	1.83	3	1.85	4	1.87	4	1.85	6	1.86
0.01	0.02	9	2.17	34	2.17	49	2.17	65	2.17	78	2.17	90	2.17	116	2.17
	0.03	4	2.06	13	2.08	19	2.07	25	2.08	29	2.08	34	2.08	44	2.08
	0.04	2	2	8	2.01	11	2	15	2.01	18	2	21	2.01	26	2
	0.05	2	2	6	1.93	8	1.94	11	1.93	13	1.94	15	1.95	19	1.94
	0.1	2	1.97	3	1.76	4	1.71	5	1.73	6	1.74	7	1.71	8	1.74
	0.15	2	1.45	2	1.56	3	1.69	3	1.6	4	1.6	4	1.6	6	1.59
	0.2	2	1.57	2	1.35	2	1.44	3	1.47	3	1.45	4	1.47	4	1.49
0.03	0.06	7	1.7	24	1.7	35	1.7	46	1.7	54	1.7	63	1.7	82	1.7
	0.09	3	1.58	9	1.58	13	1.57	17	1.58	20	1.58	23	1.58	30	1.58
	0.12	2	1.45	6	1.46	8	1.49	10	1.49	12	1.49	14	1.48	18	1.48
	0.15	2	1.34	4	1.42	6	1.39	7	1.41	9	1.41	10	1.41	13	1.41
	0.3	2	0.97	2	1.11	2	1.12	3	1.09	4	1.08	4	1.15	5	1.13
0.05	0.1	5	1.44	19	1.44	28	1.44	37	1.44	45	1.44	51	1.44	66	1.44
	0.15	2	1.3	7	1.3	10	1.3	13	1.3	16	1.31	18	1.3	24	1.3
	0.2	2	1.29	4	1.2	6	1.19	8	1.2	9	1.2	11	1.19	14	1.2
	0.25	2	0.94	3	1.1	4	1.11	6	1.1	7	1.07	8	1.07	10	1.12
	0.5	2	0.57	2	0.82	2	0.77	2	0.77	3	0.78	3	0.71	4	0.67

The presented plan is turned into the existing plan when ${{{\rho }_{uv}=\rho }_{xy}=\rho }_{yx}=\rho =0$ then becomes a special case and in this case ${\overline{Y} }_{DID}=\overline{Y }$

13 $${E(\overline{Y} }_{DID})=E\left(Y\right)=\mu $$

14 $${Var(\overline{Y} }_{DID})=Var\left(\overline{Y }\right)=\frac{{\sigma }^{2}}{n}$$

**Case 2** In an unknown circumstance.

Similar to real-world scenarios, the majority of the time the $\sigma$ is unknown and can be calculated using the sample standard deviation $(S)$. With the following steps, we suggest the sampling strategy for the $\sigma$ unknown situation in adaptation:

**Step 1** Take a random sample of size *n* from a lot and obtain the mean characteristic $\overline{y }$. Then, calculate the following statistics:

15 $$S=\sqrt{\frac{\sum_{i=1}^{n}{\left({y}_{i}-\overline{y }\right)}^{2}}{n-1}}$$

and

16 $${E}^{*}=\frac{USL-{\overline{Y} }_{DID}}{S}$$

**Step 2** accept the lot if ${E}^{*}\ge k$, otherwise reject the lot.

The following is the derivation of the OC curve function for the acceptance probability/likelihood of the suggested plan:

17 $${L}^{*}\left(p\right)=P({E}^{*}\ge k)$$

Now, according to^37^:

18 $${\overline{Y} }_{DID}+kS\sim N\left({\mu }_{y}+kE\left(S\right),Var\left({\overline{Y} }_{DID}\right)+{k}^{2}Var\left(S\right)\right)$$

We know that

19 $$ E\left( S \right) = c_{4} \sigma \;{\text{and}}\;V\left( S \right) = \sigma^{2} \left( {1 - c_{4}^{2} } \right) $$

where

20 $${c}_{4}=[2/\left(n-1\right){]}^\frac{1}{2}\Gamma (n/2)/\Gamma [(n-1)/2]$$

So,

21 $${\overline{Y} }_{DID}+kS\sim N({\mu }_{y}+k{c}_{4}{\sigma }_{x}, {\sigma }_{y}^{2}\left(\frac{1}{n\left(1-{\rho }_{xz}^{2}\right)} \left[1-{\rho }_{yx}^{2}-{\rho }_{yz}^{2}-{\rho }_{xz}^{2}+{2\rho }_{yx}{\rho }_{yz}{\rho }_{xz} \right]+{k}^{2}\left(1-{c}_{4}^{2}\right)\right)$$

Equation (4) can be used to obtain the OC function, which is as follows:

22 $${L}^{*}\left(p\right)=P\left[Z\le \frac{{Z}_{p}-k{c}_{4}}{\sqrt{\left(\frac{1}{n\left(1-{\rho }_{xz}^{2}\right)} \left[1-{\rho }_{yx}^{2}-{\rho }_{yz}^{2}-{\rho }_{xz}^{2}+{2\rho }_{yx}{\rho }_{yz}{\rho }_{xz} \right]+{k}^{2}\left(1-{c}_{4}^{2}\right)\right)}}\right]$$

Lastly, in the case where σ is unknown, the OC function can be expressed as follows:

23 $${L}^{*}\left(p\right)=\Phi \left(\frac{{Z}_{p}-k{c}_{4}}{\sqrt{\left(\frac{1}{n\left(1-{\rho }_{xz}^{2}\right)} \left[1-{\rho }_{yx}^{2}-{\rho }_{yz}^{2}-{\rho }_{xz}^{2}+{2\rho }_{yx}{\rho }_{yz}{\rho }_{xz} \right]+{k}^{2}\left(1-{c}_{4}^{2}\right)\right)}}\right)$$

As in case 1, the underlying optimization conditions will be followed to determine the plan parameters. The plan equations are subjected to the following constraints to minimize $n$:

24 $${L}^{*}\left({p}_{1}\right)=\Phi \left(\frac{{Z}_{p}-k{c}_{4}}{\sqrt{(\frac{1}{n\left(1-{\rho }_{xz}^{2}\right)} \left[1-{\rho }_{yx}^{2}-{\rho }_{yz}^{2}-{\rho }_{xz}^{2}+{2\rho }_{yx}{\rho }_{yz}{\rho }_{xz} \right]+{k}^{2}(1-{c}_{4}^{2}))}}\right)\ge 1-\alpha $$

and

25 $${L}^{*}\left({p}_{2}\right)=\Phi \left(\frac{{Z}_{p}-k{c}_{4}}{\sqrt{(\frac{1}{n\left(1-{\rho }_{xz}^{2}\right)} \left[1-{\rho }_{yx}^{2}-{\rho }_{yz}^{2}-{\rho }_{xz}^{2}+{2\rho }_{yx}{\rho }_{yz}{\rho }_{xz} \right]+{k}^{2}(1-{c}_{4}^{2}))}}\right)\le \beta $$

The values of $n$ and $k$ are found for various combinations of *AQL* and *LQL* and different values of $\rho$, like the case when $\sigma$ is known. We saw the corresponding tendency from Table 3, which is explained below:

1. For the fixed values of ${p}_{1}$, sample size $n$ decreases as ${p}_{2}$ increases. For instance, ${p}_{1}= 0.001$ at $\rho =0.7$ for ${p}_{2} = 0.004,$ $n=201; \mathrm{for }{p}_{2} = 0.006, n=110$*;* for ${p}_{2} = 0.008,$ $n=77$. But $n = 30$ for ${p}_{2}= 0.020$.
2. For the fixed values of ${p}_{2}$, sample size $n$ increases as ${p}_{1}$ decreases. For instance, ${p}_{2}= 0.020$ at $\rho =0.7$ for ${p}_{1} = 0.001,$ $n=30; \mathrm{for }{p}_{1} = 0.0025, n=51$*;* for ${p}_{1} = 0.005,$ $n=97$.
3. As the value of the $\rho$ decreases the value of sample size increases. For instance, at ${p}_{1}= 0.001$ and ${p}_{2} = 0.006$ at $\rho =0.9$ the $n=103; \mathrm{at }\rho =0.7\mathrm{ the } n=110$ and at $\rho =0.5$ the $n=117.$

**Table 3:** Plan parameters of single sampling plan using DID estimator when $\sigma$ is unknown at different values of ${{{\rho }_{xz}=\rho }_{xy}=\rho }_{yz}=\rho$.

${p}_{1}$	${p}_{2}$	$\rho =0.9$		$\rho =0.8$		$\rho =0.7$		$\rho =0.6$		$\rho =0.5$		$\rho =0.4$		$\rho =0$	
		$n$	$k$	$n$	$k$	$n$	$k$	$n$	$k$	$n$	$k$	$n$	$k$	$n$	$k$
0.001	0.002	–	–	–	–	–	–	–	–	–	–	–	–	–	–
	0.003	–	–	–	–	–	–	–	–	–	–	–	–	–	–
	0.004	189	2.84	195	2.85	201	2.85	207	2.85	212	2.85	217	2.9	227	2.9
	0.006	103	2.77	107	2.77	110	2.77	114	2.77	117	2.77	119	2.8	125	2.8
	0.008	72	2.71	75	2.72	77	2.72	79	2.72	82	2.72	83	2.7	88	2.7
	0.01	56	2.67	58	2.67	60	2.67	62	2.67	63	2.67	65	2.7	68	2.7
	0.015	37	2.59	38	2.59	39	2.59	41	2.59	42	2.59	43	2.6	45	2.6
	0.02	28	2.52	29	2.53	30	2.53	31	2.53	32	2.53	33	2.5	34	2.5
0.0025	0.005	–	–	–	–	–	–	–	–	–	–	–	–	–	–
	0.01	126	2.54	132	2.54	137	2.54	141	2.54	146	2.54	149	2.5	158	2.5
	0.015	68	2.45	71	2.46	74	2.46	76	2.46	79	2.46	81	2.5	86	2.5
	0.02	47	2.39	49	2.4	51	2.39	53	2.4	54	2.39	56	2.4	60	2.4
	0.025	36	2.34	38	2.35	39	2.34	41	2.35	42	2.34	43	2.3	46	2.3
	0.03	29	2.30	31	2.3	32	2.31	33	2.3	34	2.3	35	2.3	37	2.3
	0.05	17	2.18	18	2.18	19	2.18	20	2.19	20	2.18	21	2.2	22	2.2
0.005	0.01	–	–	–	–	–	–	–	–	–	–	–	–	–	–
	0.015	153	2.35	160	2.35	167	2.35	174	2.35	180	2.35	186	2.4	197	2.4
	0.02	88	2.28	93	2.29	97	2.29	101	2.29	104	2.29	108	2.3	115	2.3
	0.03	47	2.19	49	2.2	52	2.2	54	2.2	56	2.2	58	2.2	61	2.2
	0.04	32	2.12	33	2.13	35	2.13	37	2.12	38	2.13	39	2.1	42	2.1
	0.05	24	2.07	25	2.07	27	2.07	28	2.07	29	2.07	30	2.1	32	2.1
	0.1	11	1.88	12	1.88	12	1.89	13	1.88	14	1.86	14	1.9	15	1.9
0.01	0.02	–	–	–	–	–	–	–	–	–	–	–	–	–	–
	0.03	101	2.08	107	2.08	113	2.08	118	2.08	123	2.08	128	2.1	138	2.1
	0.04	57	2.01	61	2.01	64	2.01	68	2.01	71	2.01	73	2	79	2
	0.05	39	1.95	42	1.96	44	1.95	46	1.95	49	1.96	50	2	55	2
	0.1	15	1.76	16	1.75	17	1.76	18	1.75	18	1.76	19	1.8	21	1.8
	0.15	9	1.63	10	1.63	10	1.65	11	1.63	11	1.64	12	1.6	13	1.6
	0.2	6	1.56	7	1.54	7	1.56	8	1.55	8	1.54	9	1.6	9	1.5
0.03	0.06	130	1.70	141	1.7	152	1.7	162	1.7	171	1.7	180	1.7	198	1.7
	0.09	43	1.58	47	1.59	50	1.59	54	1.59	57	1.58	61	1.6	67	1.6
	0.12	23	1.50	25	1.5	28	1.49	30	1.5	32	1.5	33	1.5	37	1.5
	0.15	15	1.43	17	1.43	18	1.43	20	1.42	21	1.42	22	1.4	25	1.4
	0.3	5	1.13	6	1.22	6	1.19	7	1.12	7	1.17	8	1.2	9	1.2
0.05	0.1	79	1.44	88	1.44	96	1.44	105	1.44	112	1.44	119	1.4	134	1.4
	0.15	25	1.31	28	1.31	31	1.32	34	1.31	36	1.31	39	1.3	44	1.3
	0.2	13	1.21	15	1.21	16	1.21	18	1.21	19	1.21	21	1.2	24	1.2
	0.25	8	1.13	9	1.13	11	1.13	12	1.13	13	1.13	14	1.1	16	1.1
	0.5	3	0.9	3	0.73	3	0.81	4	0.65	4	0.75	4	0.8	5	0.7

## Algorithm

The proposed plan design parameters are *n* and *k* under the single sampling scheme whose detail is described in section "Design". The algorithmic steps to elaborate the flow of computing the proposed plan parameters are as follows (Fig. 1 explains the algorithm via a flow-chart):

*Step 1* Specify the values of ${p}_{1}$, ${p}_{2}$ and $\rho$.

*Step 2* Generate 100,000 values of *n* and *k* from the uniform distribution.

*Step 3* Computation of values of OC functions against ${p}_{1}$, ${p}_{2}$ and $\rho$.

*Step 4* Find out the values of *n* and corresponding *k* that satisfy the plan equations.

*Step 5* Find out the smallest values of *n* and corresponding *k* obtained in Step 4.

*Step 6* The least possible value of *n* and *k* is obtained for 100,000 times simulation results.

*Step 7* Choose the lowest value of *n* and corresponding *k* as the computed values from Step 6.

The concept needs to be addressed with theoretical significance and practical contributions which are provided as follows:

## Theoretical significance

1. *Increased Precision* Using three auxiliary variables in an estimator can enhance precision by incorporating more information, leading to more reliable and accurate estimates.
2. *Bias Reduction* The inclusion of three auxiliary variables may reduce bias, making the estimator more robust and less susceptible to biases inherent in simpler models as discussed by … for developing the three auxiliary variables-based estimator to estimate the study variable.
3. *Model Flexibility* Three auxiliary variables provide greater flexibility in modeling complex relationships, allowing for a more nuanced understanding of the underlying dynamics. That’s why researchers are making their focus to use auxiliary information-based estimators rather than utilizing complex sampling procedures which will compromise the lot sentencing cost as well.

## Practical contribution

1. *Improved Predictive Power* The use of three auxiliary variables can enhance predictive modeling, contributing to better predictions of the target variable.
2. *Variable Selection* The three auxiliary variables can aid in identifying relevant factors, contributing to better-informed decision-making and understanding of the studied system.
3. *Generalization to Multivariate Cases* Having three auxiliary variables is crucial for extending models to multivariate scenarios, capturing interactions between multiple variables.

In summary, employing an estimator with three auxiliary variables offers theoretical advantages such as increased precision and reduced bias, while practical contributions include improved predictive power, better variable selection, and the ability to handle more complex, multivariate situations.

## Results discussion

In this paper, an attempt has been made to offer a comparison picture with the approximation approach as far as the acceptance sample plans are concerned, since, to the best of the authors' knowledge, no comparable method exists. In addition, the comparative study is provided with two auxiliary information-based acceptance sampling plans, as previously mentioned. This is because no such effort has been made in the literature for the three variables usage to estimate the study variable as provided in the proposed design.

**Figure 2:**
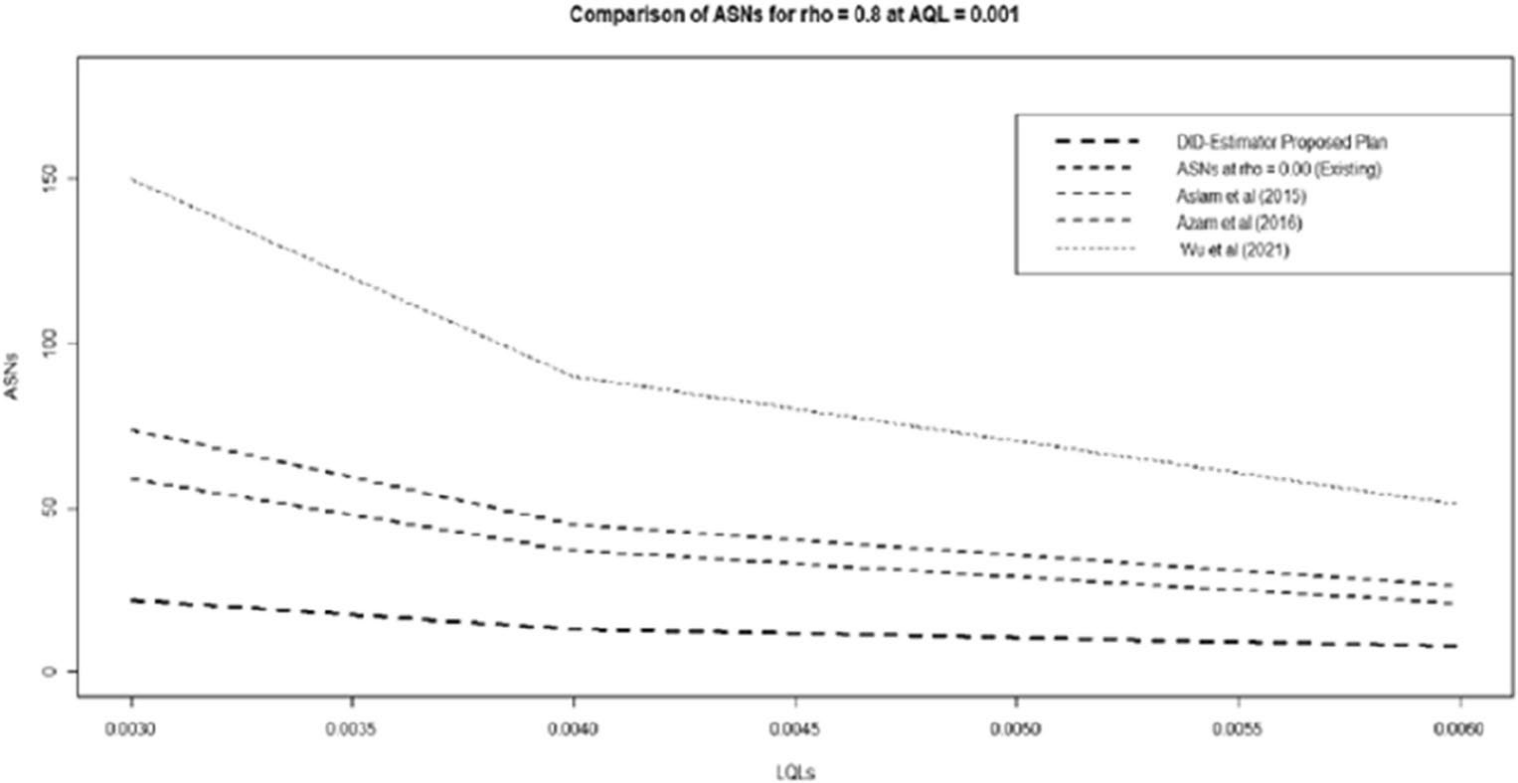
Efficiency Comparison of Existing Plans with Proposed DID Sampling Plan.

This section is divided into three sections: the specific case of the suggested plan, which explains its superiority over the existing sample plans in the literature, and a comparison study with the^38^ and^39^. Furthermore, it was discovered that the suggested sampling plan worked better than the current sampling plans, such as^38,39^, and the current sampling plans under the unique situation when ρ = 0. Figure 2 provides a graphic summary of the comparative analysis, demonstrating how the suggested plan outperformed the current plans in every way.

The proposed concept is much more suitable than the statistical process control acceptance sampling plans as far as economic studies and quasi-experimental designs, helpful to control confounding variable conditions. The suggested method requires a smaller sample size (ASNs) to be implemented for the lot sentencing procedure, according to the graphic display. This display is built using the known scenario $\sigma =0.8$ and $AQL=0.001$ fixed. The following is a thorough discussion of the comparative analysis between each current plan and the proposed plan:

## ***A comparative study with***^38^

Firstly, a comparison has been made with the existing^38^ to prove the argument that the proposed concept outperformed the existing sampling plans. The comparative study results are shown in two respective tables for both $\sigma$ known and unknown cases, such as Tables 4 and 5. In Table 4, the results are being considered for the ${p}_{1}=0.001$ and ${p}_{2}= 0.002, 0.003, 0.004 \& 0.006$ for $\rho =0.8, 0.6 \& 0.4$. Similarly, in Table 5 the results are shown for the ${p}_{1}=0.0025$ and ${p}_{2}= 0.010, 0.015, 0.020 \& 0.025$ for $\rho =0.8, 0.6 \& 0.4$ as there is a relationship between $y$ and $x$ where $x$ is treated as a piece of auxiliary information. Hence, the findings can be explained as follows:

- For the ${p}_{1}=0.001$ and ${p}_{2}= 0.002, 0.003 \& 0.006$ at $\sigma$ known case from Table 4, the proposed plan gives $n=\mathrm{57,22} \& 8$, for $\rho =0.8$ while existing plan gives size $n=153, 59 \& 21$, whereas for $\rho =0.6$ the proposed plan gives $n=106, 42 \& 15$ while existing plan gives $n=172, 67 \& 24$. The same parametric behavior can be observed for the other parametric values as shown in Table 4.
- For the ${p}_{1}=0.0025$ and ${p}_{2}= 0.010, 0.015 \& 0.020$ for the $\sigma$ unknown case from Table 5, the proposed plan gives $n=\mathrm{132,71} \& 49$, for $\rho =0.8$ while existing gives size $n=150, 82 \& 56$, whereas for $\rho =0.6$ the proposed plan gives $n=141, 76 \& 53$ while existing plan gives $n=154, 84 \& 58$. The same can be seen for the other parametric values as shown in Table 5.

**Table 4:** Comparison of Proposed Sampling Plan with^38^ for $\sigma$ Known Case.

${p}_{1}$	${p}_{2}$	Required Sample Size $n$		
		$\rho =0.8$	$\rho =0.6$	$\rho =0.4$
	0.002	57 (153)	106 (172)	149 (183)
0.001	0.003	22 (59)	42 (67)	57 (71)
	0.004	13 (37)	25 (41)	35 (43)
	0.006	8 (21)	15 (24)	20 (25)

**Table 5:** Comparison of Proposed Sampling Plan with^38^ for $\sigma$ Un-known Case.

${p}_{1}$	${p}_{2}$	Required Sample Size $n$		
		$\rho =0.8$	$\rho =0.6$	$\rho =0.4$
	0.010	132 (150)	141 (154)	149 (156)
0.0025	0.015	71 (82)	76 (84)	81 (85)
	0.020	49 (56)	53 (58)	56 (59)
	0.025	38 (43)	41 (45)	43 (45)

To demonstrate the concept's effectiveness, two auxiliary-information-based sampling plan is compared where successive occasions were targeted to estimate the study variable. To the best of the authors' knowledge, no attempt has been made to create an acceptance sampling plan in the literature with such utility. This leads to the creation of the suggested acceptance sampling plan design. So, the presented comparison is fine to the best of the author’s stance. The new index with three auxiliary variables used in this paper methodology is novel, and the use of the difference-in-difference estimator approach produces better results in terms of the smaller sample size needed to make a better decision of lot sentencing than the other existing acceptance sampling plans now in literature.

## A comparative study with^39^

Secondly, to elaborate on the efficiency with an existing^39^ Table 6 is constructed to represent the comparison of the required sample size (*n*) for lot sentencing between the proposed acceptance sampling plan with the^39^. It is clear from the results that the proposed sampling plan requires a smaller sample size as compared to the existing sampling plan and the variables *y, x, and z* are correlated with each other. The following trend is demonstrated by the computed results:

- For the ${p}_{1}=0.001$ and ${p}_{2}= 0.003, 0.020$ at $\sigma$ known case from Table 5, the proposed plan gives sample size $n=6 \& 2$, for $\rho =0.9$ and size $n=42 \& 5$, for $\rho =0.6$, and size $n=57 \& 7$, for $\rho =0.4$ whereas the existing plan shows $n=74 \& 8$.
- For ${p}_{1}=0.0025$ and for ${p}_{2}= 0.010, 0.020$ gives sample size $n=3 \& 2$, for $\rho =0.9$ and size $n=21 \& 9$, for $\rho =0.6$, and size $n=29 \& 12$, for $\rho =0.4$ where as existing plan shows $n=38 \& 16$.

**Table 6:** Comparison of Proposed Sampling Plan with^39^.

${p}_{1}$	${p}_{2}$	Proposed Plan with ${{{\rho }_{xz}=\rho }_{xy}=\rho }_{yz}=\rho$						Aslam et al. (2015)
		$\rho =0.8$	$\rho =0.8$	$\rho =0.7$	$\rho =0.6$	$\rho =0.5$	$\rho =0.4$	
0.001	0.002	15	57	83	106	128	149	194
	0.003	6	22	32	42	49	57	74
	0.004	4	13	20	25	31	35	45
	0.006	2	8	11	15	18	20	26
	0.008	2	6	8	11	13	15	19
	0.01	2	5	7	9	10	12	15
	0.015	2	3	5	6	7	8	11
	0.02	2	3	4	5	6	7	8
0.0025	0.005	13	48	69	89	108	125	162
	0.01	3	11	16	21	25	29	38
	0.015	2	7	9	12	15	17	22
	0.02	2	5	7	9	11	12	16
	0.025	2	4	6	7	9	10	12
	0.03	2	3	5	6	7	8	11
	0.05	2	2	3	4	5	5	7
0.005	0.01	11	43	59	77	93	109	141
	0.015	4	16	23	30	35	41	53
	0.02	3	10	14	18	22	25	32
	0.03	2	6	8	10	12	14	18
	0.04	2	4	6	7	9	10	13
	0.05	2	3	5	6	7	8	10
	0.1	2	2	3	3	4	4	6
0.01	0.02	9	34	49	65	78	90	116
	0.03	4	13	19	25	29	34	44
	0.04	2	8	11	15	18	21	27
	0.05	2	6	8	11	13	15	19
	0.1	2	3	4	5	6	7	8
	0.15	2	2	3	3	4	4	6
	0.2	2	2	2	3	3	4	4
0.03	0.06	7	24	35	46	54	63	84
	0.09	3	9	13	17	20	23	30
	0.12	2	6	8	10	12	14	18
	0.15	2	4	6	7	9	10	13
	0.3	2	2	2	3	4	4	5
0.05	0.1	5	19	28	37	45	51	66
	0.15	2	7	10	13	16	18	24
	0.2	2	4	6	8	9	11	14
	0.25	2	3	4	6	7	8	10
	0.5	2	2	2	2	3	3	4

## A comparative study with the sampling plan by^35^

Thirdly, the computed results have also shown that the proposed design of the sampling plan is much better than a skip-lot sampling design presented by the^35^. It can be viewed by the tabular results that the proposed plan is providing a minimal sample size in both the $\sigma$ known case (mention in Fig. 2 and Table 7). The following trend is observed:

- For the ${p}_{1}=0.001$ and ${p}_{2}= 0.003, \mathrm{0.004,0.006}$ from Table 6, the existing plan^35^ gives sample size $n=150, 90, \& 51$, for $f=0.05$ and the proposed plan gives sample size $n=22, 13, \& 8$, for $\rho =0.8$, and size $n=57, 35, \& 20$, for $\rho =0.4$.

**Table 7:** Comparison of Proposed sampling plan with^35^.

${p}_{1}$	${p}_{2}$	Proposed Plan with ${{{\rho }_{xz}=\rho }_{xy}=\rho }_{yz}=\rho$						Wu et al. (2021)
		$\rho =0.9$	$\rho =0.8$	$\rho =0.7$	$\rho =0.6$	$\rho =0.5$	$\rho =0.4$	
0.001	0.003	6	22	32	42	49	57	150
	0.004	4	13	20	25	31	35	90
	0.006	2	8	11	15	18	20	51

Hence, the proposed DID-Estimator-based sampling plan is much more efficient to use than the existing sampling plans.

## A comparative study with sampling plan for ${\varvec{\rho}}=0.0$ (a special case)

Fourthly, as a special case of the proposed DID estimator when the ${\varvec{\rho}}$ between the variables $x, y,$ and $z$ becomes zero then it becomes the existing sampling plan and the determined results are mentioned in the last column of Tables 2, 3. The proposed plan results are compared with the results $\rho =0.0$ then it can be seen that the proposed plan is more efficient than the existing one. The analysis is explained as follows:

- For the ${p}_{1}=0.001$ and ${p}_{2}= 0.003, 0.020$ at $\sigma$ known case from Table 2, the existing plan gives sample size $n=6 \& 2$, for $\rho =0.9$ and size $n=42 \& 5$, for $\rho =0.6$, and size $n=57 \& 7$, for $\rho =0.4$ whereas the existing plan shows $n=74 \& 8$.
- For ${p}_{1}=0.0025$ and for ${p}_{2}= 0.010, 0.020$ gives $n=3 \& 2$, for $\rho =0.9$ and size $n=21 \& 9$, for $\rho =0.6$, and size $n=29 \& 12$, for $\rho =0.4$ where as the existing plan shows $n=39 \& 16$.

## Results findings

To provide a real-life application of the proposed plan a real dataset was taken from the bakery products manufacturing industry which is furnished by Mason E. Wescott with $k = 2$ predictor variables provided by^40^. The problem is to correlate the green strength (flexural strength before baking) of an electric circuit breaker chute to the hydraulic pressure used in forming them and the acid concentration. The data is given in Tables 8 and 9 (Appendices), with hydraulic pressure and green strength given in units of 10 lb/in^2^ and the acid concentration is given in percentage of the nominal rate for 20 observations. The data set normality is confirmed by Shapiro. test function using the Shapiro–Wilk test for conducting the goodness of fit test and the result gives $p=0.0879.$

**Table 8:** Parameters of the Proposed Sampling Plan when ${\rho }_{yx}=0.521, {\rho }_{yz}=0.880,{\rho }_{xz}=0.152$.

P*1* ${p}_{1}$	${p}_{2}$	$n$	$k$
0.001	0.002	14	2.97
	0.003	6	2.8976
	0.004	4	2.8599
	0.006	2	2.7568
	0.008	2	2.7253
	0.01	2	2.6282
	0.015	2	2.448
	0.02	2	2.7144
0.0025	0.005	12	2.6795
	0.01	3	2.528
	0.015	2	2.4807
	0.02	2	2.3618
	0.025	2	2.2349
	0.03	2	2.4687
	0.05	2	2.133
0.005	0.01	10	2.4355
	0.015	4	2.3448
	0.02	3	2.2819
	0.03	2	2.2493
	0.04	2	2.1861
	0.05	2	2.1539
	0.1	2	2.074
0.01	0.02	9	2.1708
	0.03	4	2.1011
	0.04	2	1.9995
	0.05	2	1.9031
	0.1	2	1.553
	0.15	2	2.0151
	0.2	2	1.4949
0.03	0.06	6	1.7005
	0.09	3	1.5501
	0.12	2	1.4184
	0.15	2	1.353
	0.3	2	1.2103
0.05	0.1	5	1.4462
	0.15	2	1.3176
	0.2	2	1.1602
	0.25	2	1.0167
	0.5	2	1.2582

**Table 9:** Arc Chute Dataset from^40^.

Green Strength ${\varvec{Y}}$ in units of 10 lb/in^2^	Hydraulic Pressure ${\varvec{X}}$ in units of 10 lb/in^2^	Acid Concentration ${\varvec{Z}}$, as % of nominal rate
665	110	116
618	119	104
620	138	94
578	130	86
682	143	110
594	133	87
722	147	114
700	142	106
681	125	107
695	135	106
664	152	98
548	118	86
620	155	87
595	128	96
740	146	120
670	132	108
640	130	104
590	112	91
570	113	92
640	120	100

The variable of interest is represented by $Y$ (Green strength in units of 10 lb/in^2^) and Auxiliary variables are $X$ (Hydraulic pressure in units of 10 ln/in^2^) and $Z$ (Acid concentration as % of nominal rate) respectively. According to the steps of the proposed sampling plan (mentioned in section methods), we proceed as follows:

**Step 1** A random sample of size $n = 5$ is selected from Table 9 computed results (constructed for the obtained values of $\rho$) against ${p}_{1} = 0.05, {p}_{2} = 0.1$ and $k = 1.4462$*.* Assuming the value of the upper specification limit as $USL = 1200$.

$${\mu }_{y}=641.6, {\mu }_{x}=131.4, {\mu }_{z}=100.6, {\rho }_{yx}=0.521, {\rho }_{yz}=0.880,{\rho }_{xz}=0.152, \overline{y }=662.9$$,

$$ \overline{x} = 135.6,\;\overline{z} = 104.2, \overline{Y}_{DID} = 1153.11 {\text{and}} E = 0.8796. $$

**Step 2** From the above results we see that $E<k$ i.e., 0.8796 < 1.4462, hence, the lot will be rejected.

Using multiple auxiliary variables was very helpful in obtaining an accurate estimate of the research variable. The sample demonstrated the strength of the suggested acceptance sampling approach, showing that even with a small sample size of five units, the plan may provide accurate results by assisting the researchers in determining the lot sentencing procedure.

## Conclusions

Using the DID estimator developed by^36^, a single sample-based acceptance sampling strategy has been put forth in this paper. The assumption that the product inspection lifetime follows a normal distribution guides the study of the dependent variable together with two independent variables. This study creates an acceptance sampling strategy to implement the^36^ in a real-world industrial setting for quality assurance through SPC tools. The suggested plan OC function has been designed, and the simulation process's parametric outcomes are ascertained by utilizing an efficient grid search strategy to obtain the smallest possible sample size using R software. The findings are presented in detail in Tables 2, 3, where it is observed that the proposed sampling plan is more effective in providing the smaller sample size value needed for lot sentencing, while the flow chart and algorithm provide more information about the application and computations of the proposed plan. The results clearly show the significant superiority of the proposed plan over the current^35,38,39^, and the special case of the correlation zero. Furthermore, there is a good chance that the suggested strategy will be applied to other fields utilizing various sample schemes in the future. Other sectors can benefit more from implementing the DID estimator-based sample plan as it is evident that it is more efficient than the current sampling plans. Future research can examine the suggested study's use of ranked set sampling, multiple dependent state sampling, and repeating sampling.

## Data Availability

The data is given in the paper.

## Appendix

See Tables 8, 9.
